# Being watched by others eliminates the effect of emotional arousal on inhibitory control

**DOI:** 10.3389/fpsyg.2015.00004

**Published:** 2015-01-20

**Authors:** Jiaxin Yu, Philip Tseng, Neil G. Muggleton, Chi-Hung Juan

**Affiliations:** ^1^Institute of Neuroscience, National Yang-Ming University, Taipei, Taiwan; ^2^Institute of Cognitive Neuroscience, National Central University, Jhongli, Taiwan; ^3^Brain and Consciousness Research Center, Taipei Medical University – Shuang Ho Hospital, New Taipei City, Taiwan; ^4^Institute of Cognitive Neuroscience, University College London, London, UK; ^5^Department of Psychology, Goldsmiths, University of London, London, UK

**Keywords:** cognitive control, conscientiousness, emotion regulation

## Abstract

The psychological effect of being watched by others has been proven a powerful tool in modulating social behaviors (e.g., charitable giving) and altering cognitive performance (e.g., visual search). Here we tested whether such awareness would affect one of the core elements of human cognition: emotional processing and impulse control. Using an emotion stop-signal paradigm, we found that viewing emotionally-arousing erotic images before attempting to inhibit a motor response impaired participants’ inhibition ability, but such an impairing effect was completely eliminated when participants were led to believe that their facial expressions were monitored by a webcam. Furthermore, there was no post-error slowing in any of the conditions, thus these results cannot be explained by a deliberate speed-accuracy tradeoff or other types of conscious shift in strategy. Together, these findings demonstrate that the interaction between emotional arousal and impulse control can be dependent on one’s state of self-consciousness. Furthermore, this study also highlights the effect that the mere presence of the experimenter may have on participants’ cognitive performance, even if it’s only a webcam.

## INTRODUCTION

The psychological effect of being watched by others has been proven a powerful tool in boosting honest or charitable behaviors (Bateson et al., 2006; Ekstrom, 2012) while reducing dishonest behaviors (Nettle et al., 2012). This watching effect increases self-awareness (Baltazar et al., 2014) and causes individual to consciously modify their behavior to increase compliance with social standards (Wedekind and Milinski, 2000; Milinski et al., 2002). However, besides these high-level socio-behavioral changes that are subject to participants’ conscious decisions, it remains unclear whether the effect of watchful eyes can modulate some of the core elements of human cognition, such as cognitive control and emotion regulation. Several studies in social psychology have revealed that the Stroop interference effect can be significantly reduced if the experimenter looks frequently at the participants during the task (i.e., Huguet et al., 1999). This is known as the “social facilitation-inhibition” effect, where social context inhibits automatic and purpose-irrelevant stimulus (i.e., word meaning) and leads to performance enhancement (speed of color naming). In addition to Stroop interference, one recent study using visual search has also shown that people may alter their cognitive performance by searching slowly in order to achieve higher accuracy rates when they are being (or believe they are) watched by others (Miyazaki, 2013).

This type of facilitation effect could be potentially valuable if it can be applied to cognitive and emotional control since training programs to improve performance usually take a long period of time (e.g., Berkman et al., 2014), and the uses of drugs (Li et al., 2010) or brain stimulation techniques (Hsu et al., 2011) can raise ethical issues. As such, in this study we explored whether increased levels of conscientiousness, or the knowledge of being watched by others, may influence the effects that emotion has on cognitive control.

Studies investigating cognitive processes behind inhibitory control have shown that seeing emotional stimuli prior to performing a stop-signal task, which assesses the efficiency in inhibiting planned responses (Verbruggen and Logan, 2008), can significantly impair inhibitory control (Verbruggen and De Houwer, 2007) by prolonging participants’ time to stop a preplanned response (stop-signal reaction time, SSRT). This is because the emotional stimuli elicit a response in participants (whether they like it or not) and therefore delay the onset of the actual inhibition processes. Recently, by using emotional stimuli of matched arousal and valence levels between female and male participants, Yu et al. (2012) found that erotic and painful stimuli impaired male participants’ ability to inhibit motor responses, but not females. This finding suggests that, even when arousal and valence levels are equated between both genders, men in general suffer greater impairment in inhibitory control ability in the face of emotional stimuli or events. This was especially true when stimuli were erotic images (Yu et al., 2012). In light of these findings, here we adopted the same erotic pictures and stop-signal task in a naïve group of male participants to investigate whether the awareness of being watched by others would interact with the known effects of emotional stimuli on inhibitory control. Although we did not anticipate any effect of emotional arousal on females’ inhibitory control in this experimental context, as a control condition, we also tested the same paradigm in a group of naïve female participants (Control Experiment 2).

The emotional stop-signal paradigm here was coupled with the manipulation of being watched by the experimenter. This manipulation was achieved by turning a webcam ON (being watched) or OFF (not being watched) in front of the participants, which should induce higher levels of conscientiousness in them (Javaras et al., 2012). An increase (i.e., worse performance) in the already-impaired SSRT would imply that being-watched has an additional negative impact over and above erotic pictures on men’s inhibitory control, whereas a decrease in SSRT (i.e., better performance) would suggest improved inhibition; both of which would demonstrate a link between emotion and the effect of being-watched. Alternatively, a null effect would predict no additional changes to the emotion-driven impairment in SSRT. Hence, to compare the effect of being-watched in a more appropriate context, we also conducted a control experiment that examined the effect of being-watched in a classic stop-signal task (i.e., no erotic pictures).

Additionally, the control adjustment process was analyzed to evaluate the effects of camera and emotion. Post-error slowing was used as an index of strategy adjustment to assess whether individuals were prolonging their response time to make less errors (Li et al., 2008). If the awareness of being-watched affects participants’ conscious decision boundary, we should observe increased post-error slowing RTs in all experimental conditions regardless of our manipulation of conscientiousness.

## MATERIALS AND METHODS

### PARTICIPANTS AND RECRUITMENT

To make sure all participants understood the stop-signal task, they were required to pass a prescreen stop-signal task before being recruited. There were 72 go trials and 24 stop trials in the prescreen test, and all participants needed to meet two criteria (1) the go accuracy rate was higher than 95%, and (2) the stop-response rate was between 45 and 55%. Forty-two male participants passed the prescreen test and took part in this study, 26 of which (21.6 years, from 18 to 27) were randomly assigned to the main experiment, and 16 (22.4 years, from 18 to 29) to the control experiment. All experimental procedures were approved by the Institutional Review Board of National Taiwan University, Taipei, Taiwan.

### APPARATUS

The visual stimuli were presented on a 23-inch LCD monitor. Stimulus presentation and data acquisition were done using Matlab with Psychtoolbox-3. To manipulate being watched or not, we placed one web camera (Microsoft LifeCam HD-3000) on top of the monitor.

### DESIGN AND PROCEDURES

#### Main Experiment

Participants were randomly assigned to the ON or OFF group at the start of this experiment. Participants in these two groups were given the same instructions regarding the stop-signal task. An additional sentence was included in the ON group instruction: “during this experiment, your facial expressions will be recorded and will be analyzed after you finish the whole experiment^1^,” and the recording program would be opened in front of them momentarily to convince them that we were recording their facial expressions. All participants were told that the experimenter would be absent from the room until they finished the whole session.

**FIGURE 1:**
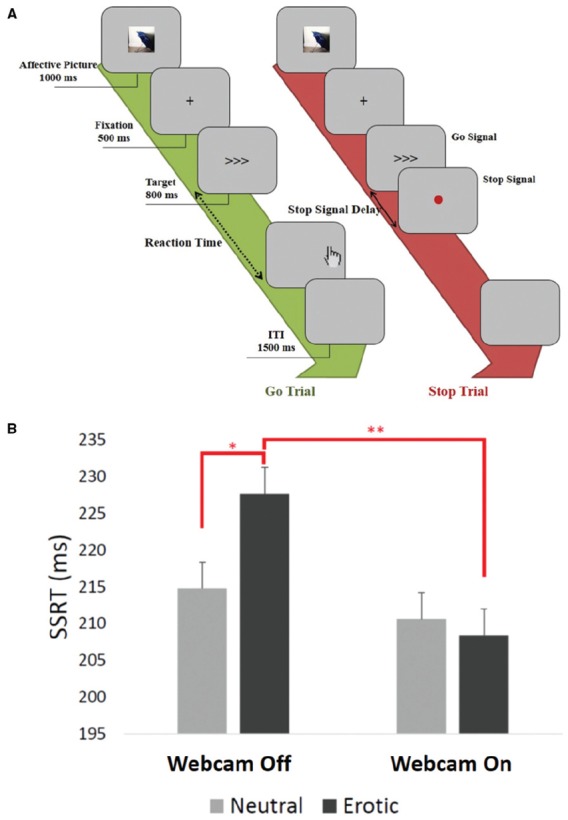
**(A)** After an emotional stimulus, participants needed to do a speeded choice response task in which they were required to withhold response if a stop signal was presented. There were 144 go trials and 48 stop trials in each emotion condition. One erotic/neutral image was shown to participants shortly before each trial for 1 s. **(B)** Mean of stop-signal reaction times (SSRTs), with 95% confidence interval error bars. No difference was seen in SSRTs in the neutral condition (*F* = 0.137, *p* = 0.715) but a significant difference in SSRTs in the erotic condition (*F* = 8.540, *p* = 0.007) was seen between the two groups. *p < 0.05, **p < 0.01.

For the stop-signal task, participants were to perform a speeded choice response task and were to withhold that response if a stop signal was presented. Participants’ were required to press the left or right button of response box with their left or right index finger according to the direction of the arrow (go signal; Figure 1). In 75% of the trials, this made up the entire trial (go trials). In the remaining 25% of trials, a red dot (stop signal) appeared shortly after the go signal, prompting the participants to withhold their go response (Figure 1). Since the paradigm was set to adjust difficulty by altering the time of onset of the stop signal (stop-signal delay, SSD) with a staircase procedure, the obtained measures, SSD and go reaction time, as well as their difference (SSRT), provided an estimate of participants’ ability to inhibit a prepotent response. All participants completed an emotional stop-signal task, including two erotic and two neutral blocks, on the same day in counterbalanced order. In the emotional stop-signal task, there were 144 go and 48 stop trials in each condition. One erotic or neutral image was shown to the participants shortly before each trial for 1 s (Figure 1A). For image stimuli, 48 erotic and 48 neutral images (picture size: 300 × 300 pixels) were retrieved from the International Affective Picture System (IAPS). It was ensured that the images differed in terms of their valence (erotic: mean = 7.2, standard error (SE) = 0.74; neutral: mean = 5.0, SE = 0.4) and arousal (erotic: mean = 6.5, SE = 0.89; neutral: mean = 2.73, SE = 0.67). More details, as well as all the images used in the present study, can be found in the study by Yu et al. (2012).

#### Control Experiment 1

The purpose of the control experiment was twofold: (1) to assess the effects of being-watched on a classic stop-signal task (i.e., no erotic pictures), and (2) use a within-subject design to better control for any possible group differences that might be present in the main experiment. All participants completed a classic stop-signal task (without any emotional pictures, 144 go and 48 stop trials in each ON/OFF condition), including ON and OFF conditions in a group-counterbalanced order. All experimental procedures and materials were identical to the main experiment. Note that to avoid any possible gender interaction, the male experimenter in the main and control experiments was the same.

#### Control Experiment 2

Although our previous research has suggested that female participants are not affected by the emotion stop-signal manipulation, it was still necessary to check for any gender differences since we added a new webcam manipulation. In this control experiment, a female version of the task (female experimenter and female participants) was conducted for comparison with the males’ results. Twelve female participants were randomly assigned to the camera ON or OFF group. All experimental procedures and materials were identical to the main experiment.

#### Data analysis

Data analysis was conducted using Matlab (Mathworks) software for both experiments. The go RTs were filtered by removing incorrect trials. Trials with latencies more than two standard deviations from each participant’s mean go RT for each emotional condition were also excluded. Each participant’s critical SSD was calculated by averaging the stop signal durations of all stop trials. The SSRT was calculated using each participant’s mean go RT and subtracting the critical SSD. Mixed model repeated measures analysis was carried out for correct go RT and SSRT under the two emotion conditions between the two groups in the main experiment. To ensure that there were no preexisting differences between participants that were randomly assigned to the watched/unwatched groups, an additional one-way ANOVA was applied to test differences in SSRTs in the neutral condition between these two groups of participants. Paired *t*-tests were used to compare both correct go RTs and SSRTs for ON and OFF conditions in the control experiment.

For post-error slowing, successful go trials were categorized into two types: go trials after a correct go trial (pG) and go trials after a stop-error trial (pSE). In the present study, the difference in RTs between pSE and pG served as an index of control adjustment, which might signify a deliberate and strategic slowing by the participants to increase the likelihood of successful stopping following an error.

## RESULTS

### MAIN EXPERIMENT

For SSRTs, a main effect of emotion condition [*F*_(1,24)_ = 15.434, *p* < 0.001], no effect of being watched [*F*_(1,24)_ = 1.488, *p* = 0.23], and a significant interaction between these two factors [*F*_(1,24)_ = 6.397, *p* = 0.02] was observed. Paired *t*-tests showed a significantly slower SSRT only in the unwatched groups when viewing erotic pictures, compared to the neutral pictures (*t* = 2.971, *p* = 0.01), with no difference in the watched group (*t* = –0.739, *p* = 0.47). No difference in SSRTs in the neutral condition (*F* = 0.137, *p* = 0.72] was seen, but a significant difference in SSRTs in the erotic condition (*F* = 8.540, *p* < 0.01) was observed between the two groups (Figure 1B). For Go RTs, there were no significant effects of emotion condition [*F*_(1,24)_ = 0.005, *p* = 0.95], being watched [*F*_(1,24)_ = 0.936, *p* = 0.34], or interaction of the two factors [*F*_(1,24)_ = 2.106, *p* = 0.16]. For post-error slowing, there was no main effect of emotion condition [*F*_(1,24)_ = 1.432, *p* = 0.24] or being watched [*F*_(1,24)_ = 3.633, *p* = 0.07], and also no interaction [*F*_(1,24)_ = 0.031, *p* = 0.86] was observed. All the results are summarized in Table 1.

**Table 1:** Summary of the main experiment results (mean ± standard error).

**Main experiment**	**Webcam off**		**Webcam on**	
	**Neutral**	**Erotic**	**Neutral**	**Erotic**
Go-trial accuracy (%)	99.1 ± 0.3	98.4 ± 0.3	99.4 ± 0.3	99.4 ± 0.3
Mean go RT (ms)	408.3 ± 19.2	398.0 ± 21.8	376.7 ± 11.2	386.1 ± 14.8
SSD (ms)	193.5 ± 21.7	170.3 ± 21.8	166.1 ± 10.7	177.7 ± 15.4
SSRT (ms)	214.8 ± 3.9	227.6 ± 2.4	210.6 ± 4.3	208.4 ± 4.5
Non-cancel rate (%)	47.6 ± 0.8	48.9 ± 0.9	48.1 ± 0.8	48.6 ± 0.6
Post-stop-inhibit RT (ms)	418.6 ± 16.6	406.7 ± 21.1	394.3 ±10.5	397.3 ± 12.8
Post-stop-error RT (ms)	428.2 ± 18.2	416.3 ± 19.6	395.5 ±11.4	400.8 ± 14.2
Post-go RT (ms)	397.8 ± 16.2	392.4 ± 19.3	380.2 ±11.0	390.2 ± 13.6

### CONTROL EXPERIMENT 1

Paired *t*-tests showed no difference between ON and OFF conditions for Go RTs (*t* = –0.379, *p* = 0.71), SSRTs (*t* = 0.153, *p* = 0.88), or post-error slowing (*t* = 0.036, *p* = 0.97). Therefore, the control experiment suggests that (1) there is no additional effect of being watched on the processes of motor inhibition, and (2) the marginally significant effect of post-error slowing in the being-watched condition from Experiment 1 is likely reflective of between-subject group differences, which were no longer present when a within-subject designed was used. All the results are summarized in Table 2.

**Table 2:** Summary of the Control Experiment 1 results (mean ± standard error).

**Control Experiment 1**	**Webcam off**	**Webcam on**
Go-trial accuracy (%)	99.4 ± 0.3	99.1 ± 0.3
Mean go RT (ms)	394.7 ± 14.9	391.5 ± 11.2
SSD (ms)	172.6 ± 14.3	168.2 ± 12.0
SSRT (ms)	220.5 ± 5.5	221.2 ± 4.8
Non-cancel rate (%)	48.8 ± 0.5	48.8 ± 0.8
Post-stop-inhibit RT (ms)	414.8 ± 16.0	390.3 ± 11.4
Post-stop-error RT (ms)	415.5 ± 19.2	400.3 ± 13.3
Post-go RT (ms)	398.1 ± 11.1	382.6 ± 9.2

### CONTROL EXPERIMENT 2

For SSRTs, there was no main effect of emotion [*F*_(1,10)_ = 0.284, *p* = 0.606], being watched [*F*_(1,10)_ = 0.81, *p* = 0.389], and no interaction between the two [*F*_(1,10)_ = 0.011, *p* = 0.917]. Therefore, consistent with previous findings (Yu et al., 2012), female participants were not affected by the emotion-arousal manipulation in the context of a stop-signal paradigm. Results are summarized in Table 3.

**Table 3:** Summary of the Control Experiment 2 results (mean ± standard error).

**Control Experiment 2**	**Webcam off**		**Webcam on**	
	**Neutral**	**Erotic**	**Neutral**	**Erotic**
Go-trial accuracy (%)	95.1 ± 2.2	98.2 ± 0.8	98.8 ± 0.4	99.1 ± 0.5
Mean go RT (ms)	407.5 ± 47.1	404.2 ± 38.6	446.3 ± 27.0	440.8 ± 25.4
SSD (ms)	176.0 ± 48.5	171.5 ± 32.8	226.1 ± 29.1	215.6 ± 25.9
SSRT (ms)	228.6 ± 13.0	230.9 ± 12.7	217.2 ± 7.2	220.7 ± 6.3
Non-cancel rate (%)	50.0 ± 2.5	49.0 ± 1.4	48.3 ± 0.7	47.6 ± 1.4

## DISCUSSION

Studies have demonstrated the psychological effect of being watched by others as a powerful tool in changing social behavior. Our results showed that such awareness also alters individual’s inhibitory control ability within an emotional context. From these results, there are several points to consider. First, in the condition where the observing camera is thought to be off, the present experiment replicates our previous findings (Yu et al., 2012), demonstrating that the presence of emotional stimuli such as erotic pictures can impair male participants’ ability to inhibit motor responses. Second, when the camera is thought to be on, the awareness of being watched by the experimenter eliminated the impairing effect of erotic stimuli on SSRTs. This counteracting effect cannot be explained by an overall increase in motivation that is often associated with a watchful experimenter (Miyazaki, 2013) because, if this was true, then we should have observed better SSRT in the camera ON condition of the control experiment, where erotic images were absent. Therefore, it is clear that the effect of being watched by others specifically acts on the (impairing) effect of emotion. Consequently, such monitoring eliminates the emotion-induced impairment of SSRT, but does not enhance SSRTs in general when emotion stimuli were absent.

Though acting specifically on the effect of emotion, it is important to note that the effect of being watched itself need not be an emotional one in nature. Support for this notion comes from a comparison between the effects of erotic pictures and the webcam observation. If the effect of being watched also elicits an emotional response within the participants, then the camera ON condition in the control experiment should also yield a change in SSRTs similar to the effect of erotic pictures, and perhaps even an additional and cumulative effect on SSRTs when erotic pictures and the camera observation were combined. One plausible alternative explanation for such rapid improvement in SSRT is the idea of conscientiousness (or vigilance), or the ability to maintain alertness and attentiveness over a period of time. Conscientiousness is thought to be related to effortful control (Caspi et al., 2005) and emotion regulation (Roberts et al., 2007). Although previous studies have only reported a negative correlation between conscientiousness and negative emotion recovery (i.e., Javaras et al., 2012), there is no solid evidence that conscientiousness would not down regulate high valence and high arousal emotion such as may be caused by the erotic stimuli in the male group. This attentional account would explain the dramatic improvement in inhibitory control, but would also imply a different mechanism here from the high-level, “being-watched” socio-behavioral literature reviewed above, and instead suggest that the present effect is located more on the implicit end of the conscientiousness spectrum. This idea is also supported by the lack of significant findings in our post-error slowing analysis, which suggests that participants were not, or could not, deliberately trying to slow down their response times in order to gain higher accuracy.

Participants in the being-watched group knew that their facial expressions would be assessed, which could lead them to suppress their facial expressions either consciously or unconsciously. Our results here are more supportive of the unconscious notion because, had our participants attempted to consciously suppress their expressions (which requires mental resource), such top-down control would in theory interfere with inhibitory control and consequently prolong the stopping processes (instead of decreasing SSRT in the emotion condition, and not affecting SSRTs in Control Experiment 1). In contrast, if the suppression is done unconsciously or automatically, based on the embodied approach of emotion, it could reduce participants’ experience of emotion/arousal and resulting in a null effect of emotion (Niedenthal, 2007). It seems that the idea of being watched by others most likely unconsciously triggers such expression suppression and leads to the neutralization of emotion’s impact on on-going cognitive processes. If this is true, the automatic nature of such a modulating effect possibly makes it a suitable method for improving inhibitory control in some clinical populations such as people who are substance-dependent and would benefit from enhancement of their cognitive control ability to battle cravings.

Nevertheless, irrespective of the origin of these differences, the present findings highlight the interaction between emotional arousal and cognitive inhibition processes depending on the participants’ awareness of being watched by others. In laboratory settings, these findings also raise an important issue for the field to consider: that the presence of an experimenter or monitoring device may have a profound impact on the data we collect (e.g., Miyazaki, 2013). In addition, these results also provide a possible basis for clinical use by incorporating the conscientiousness factor into cognitive training programs and treatments for people who suffer impaired cognitive control. However, it should be noted that the present study mainly tested the watching effect in the context of positive valence emotions (i.e., erotic pictures in this study) in male participants. It remains unclear whether such an effect can also be applied in the context of negative pictures or different types of emotion (e.g., anger). Also, we did not estimate individual anxiety and stress levels for the camera ON/OFF conditions, which could be a possible mediator of the effects of emotion on cognitive processes. It would be fruitful for future studies to pinpoint the cognitive processes that would benefit from a high-level of awareness of being watched, such as inhibitory control in men in this case, and take advantage of such interaction to facilitate cognitive functioning in different populations.

### Conflict of Interest Statement

The authors declare that the research was conducted in the absence of any commercial or financial relationships that could be construed as a potential conflict of interest.

## Footnotes

1 Participants were asked whether they thought the camera was recoding after they finished the experiment. All participants reported yes.
